# Effects of methamphetamine on human effort task performance are unrelated to its subjective effects

**DOI:** 10.1007/s00213-025-06853-4

**Published:** 2025-07-08

**Authors:** Evan C. Hahn, Hanna Molla, Jessica A. Cooper, Joseph DeBrosse, Harriet de Wit

**Affiliations:** 1 Department of Psychiatry and Behavioral Neuroscience, University of Chicago, 5841 S Maryland Ave MC3077, Chicago, IL USA; 2 Department of Psychiatry and Behavioral Sciences, Emory University, Atlanta, GA USA

**Keywords:** Methamphetamine, Effort-based decision-making, Reward processing, Subjective value, Computational modeling

## Abstract

**Rationale:**

Stimulant drugs increase objective indices of reward-related behavior, including willingness to expend effort for reward, and also produce feelings of well-being and positive mood. However, it is not known to what extent these different measures are related to each other.

**Objectives:**

The present study was designed to assess the relationship between the behavioral measure of effort expenditure and positive subjective responses to methamphetamine (MA).

**Methods:**

96 healthy adults completed the Effort Expenditure for Rewards Task (EEfRT) during two laboratory sessions after receiving 20 mg MA or placebo (PL) under double blind conditions. They also self-reported their mood states and drug effects.

**Results:**

MA (vs. PL) increased willingness to complete a high effort/high reward option vs. a low effort/low reward option during the EEfRT (*N* = 96), and this effect was greater in participants with low effort at baseline. A subjective value modeling analysis (*N* = 91) showed that MA decreased sensitivity to the perceived cost of effort for the low baseline performance group only. MA also increased self-reported positive affect (euphoria; *N* = 94, liking the drug; *N* = 92) in the full sample, but this increase was unrelated to either baseline EEfRT performance or MA-induced EEfRT performance changes (*N* = 91).

**Conclusions:**

As reported previously, MA increased choice of the high effort/high reward option, particularly in participants with low effort at baseline, who also showed drug-induced changes in effort sensitivity. These behavioral effects were not related to drug liking and drug-induced euphoria. These findings suggest that the effects of stimulants on reward-related behavior and mood are dissociable.

**Supplementary Information:**

The online version contains supplementary material available at 10.1007/s00213-025-06853-4.

## Introduction

Amphetamine and other stimulant drugs produce reward-related cognitive, behavioral and mood-altering effects thought to be related to dopamine (DA) function (Roberts et al. 2020; van der Schaaf et al. 2013; Webber et al. 2021). For example, stimulant drugs enhance reward-related behaviors such as reversal learning (van der Schaaf et al. 2013), reactivity to appetitive conditioned stimuli (Taylor and Robbins 1984), and willingness to exert effort (Salamone and Correa 2024). They also induce feelings of euphoria and well-being in healthy adults (Abi-Dargham et al. 2003; Webber et al. 2021). An understudied question is whether these different effects induced by stimulants are mediated by the same, or different, underlying processes. Individual differences in task performance and mood states, both in a drug free state and after administration of a DA-ergic drug, may shed light on shared and separate mechanisms. Prior research has shown that differences in DA function contribute to both drug-free performance as well as responses to stimulant drugs (Cools and D’Esposito 2011; Lott et al. 2005; Westbrook et al. 2020). In the present analysis, we examined individual differences in effects of a stimulant drug on both willingness to exert effort and mood states in healthy adults.

One prominent theory about the role of DA in reward function is that it mediates the *motivation* to obtain rewards (Salamone and Correa 2024). In humans, deficits in DA function are associated with reduced motivation and willingness to exert effort, as seen in depression and Parkinson’s Disease, and drugs that increase DA function reverse some of these deficits (Barbeau 1969; Pary et al. 2015). Reduced willingness to exert effort has been studied in laboratory animals, showing that low DA function is associated with low levels of motivation, and drugs that increase DA function, such as amphetamine and the d-amphetamine pro-drug lisdexamfetamine, increase effort-based choices (Salamone and Correa 2022; Yohn et al. 2016). Further, Cagniard et al. (2006) reported that motivation to work, but not learning, for a food reward appeared to be influenced by tonic DA activity in brain areas relevant for a reward-directed behavior. The task developed to assess willingness to exert effort in rodents (Cousins et al. 1996) has been adapted for humans in the Effort Expenditure for Rewards Task (EEfRT; Treadway et al. 2009). As in the animal studies, amphetamine (20 mg) increases the selection of the high cost/high reward option in humans during EEfRT (Bardgett et al. 2009; Wardle et al. 2011).

There is evidence that individual differences in responses to amphetamines are related to baseline performance on these tasks, and possibly baseline DA levels (Cocker et al. 2012; Cools and D’Esposito 2011). Both rodents and human volunteers vary in their baseline levels of willingness to exert effort, and these baseline differences predict response to an acute dose of amphetamine. In one study (Cocker et al. 2012), the effects of amphetamine in rats depended on the animals’ initial preference for the low or high effort options. Amphetamine increased the choice of the high effort option only in animals with low baseline preference. Similarly in human volunteers, Soder et al. (2021) found that the effects of d-amphetamine (20 mg) were related to pre-drug performance on EEfRT; that is, d-amphetamine improved effort expenditure only in participants who exerted low effort at baseline (Soder et al. 2021).

The first goal of the current study was to examine behavioral processes mediating the effects of methamphetamine (MA) on the EEfRT performance. Drugs could affect performance on the EEfRT by altering sensitivity to (i) the perceived cost of effort required for a reward, (ii) the probability of receiving reward, (iii) reward magnitude, or (iv) some combination of these aspects. In addition to their findings on baseline performance, Soder et al. (2021) found that d-amphetamine increased willingness to exert effort especially at low-to-intermediate probabilities of reward and low reward magnitudes, and reduced sensitivity to perceived cost of effort as assessed by a computational model. In the present study, we examined the effect of MA (20 mg) on several indicators of performance on the EEfRT. We examined the effects of the drug on different probabilities and magnitudes of reward, in participants with varying levels of effort at (pre-drug) baseline. The subject sample in this analysis (*N* = 96) was larger than the sample used by Soder et al. (*N* = 30), providing additional power to detect individual differences.

A second goal in this study was to examine the relationship between the effects of the drug on EEfRT performance and its mood-enhancing effects. In humans, stimulants produce feelings of well-being and euphoria, and when given a choice, most volunteers choose a stimulant over a placebo (Johanson and Uhlenhuth 1980; Lasagna et al. 1955). This is consistent with the drugs’ reinforcing effects in laboratory animals (Pickens 1968). Yet, the extent to which the cognitive and affective actions of stimulants are related to one another is not known. Some evidence has linked the feelings of euphoria to increases in DA function (Abi-Dargham et al. 2003), although others have not (Berridge and Robinson 2016; Brauer et al. 1997). The effects of stimulants may also depend on the context in which they are taken. Hoots et al. (2021) reported that the subjective and motor effects of d-amphetamine differentially predicted whether they desired to used the drug again, depending on the context of drug-taking: positive subjective effects predicted desire to use the drug again for work or studying purposes, while faster responding on the EEfRT task predicted desire to use it in a more recreational setting. Despite the dual roles of DA on cognitive and mood effects, few studies have explored the relationship between these measures. In the present study, we examined (i) individual differences in responses to 20 mg MA on willingness to exert effort for a reward in healthy adults and (ii) the relationship between on the effects of the drug on effort and its effects on positive mood.

In the present analysis, 96 healthy participants completed the EEfRT at a pre-study orientation session, then again during two laboratory sessions after receiving 20 mg MA and placebo under double blind conditions. Participants rated their mood at regular intervals during the sessions. After study completion, the participants were separated into high and low baseline effort groups by median split, based on their task performance during the orientation session. Then, a computational model of subjective value of reward was used to assess the effect of MA on sensitivity to the cost of effort required to obtain a reward and sensitivity to reward probability, first in the full group, and then within high and low baseline effort subgroups. We hypothesized that MA would increase effort expenditure preferentially in those with lower baseline performance and reduce sensitivity to the subjective cost of effort required for a reward. Furthermore, we expected that effort increases after MA would be correlated with drug-induced increases in positive subjective ratings.

## Materials and methods

### Design

The study used a within-subjects, double-blind, placebo-controlled design. Healthy adults participated in an orientation (practice) session, followed by two 4-hour laboratory sessions on which they received 20 mg MA or placebo. The sessions were separated by at least 3 days. Participants completed EEfRT during the orientation session and at the time of peak effect on both laboratory sessions. They completed standardized measures of subjective drug effects at regular intervals during the sessions.

## Participants

Healthy men and women were recruited from the Chicago community via flyers and social media advertisements. Study inclusion criteria were age 18–35 years old, right-handedness, fluency in English, minimum high school education, BMI 19–26 kg/m^2^, and no current medications aside from contraception. Screening included a physical exam, electrocardiogram, semi-structured psychiatric interview, and detailed drug use history questionnaire. Exclusion criteria were variables that might influence safety or magnitude of response to the drug, including major psychiatric conditions, severe substance use disorder, history of cardiac disease or abnormal electrocardiogram, > 4 alcoholic or caffeinated beverages a day, working night shifts, or pregnancy or planned pregnancy in women. Women not using hormonal contraception were tested only during the follicular phase of their menstrual cycle (1–12 days from menstruation) because responses to stimulant drugs are dampened during the luteal phase of the cycle (White et al. 2002). All study procedures were approved by the University of Chicago Institutional Review Board, and all participants provided written informed consent at the University of Chicago, where the study sessions were conducted. There were 113 subjects total, the final analysis presented here consisted of *N* = 96 subjects (48 men, 48 women). Participant demographics and drug use history are reported in the supplemental materials. The present report includes data from the first two sessions of a larger, 4-session study (Molla et al. 2023).

A total of 113 subjects completed the study, but 7 were excluded from this analysis because of missing data on at least one session, 5 for always or never selecting the hard option every trial during at least one session, and 5 for never making any selections (i.e. all trials were timeouts) during at least one session, leaving *N* = 96 for the behavioral analysis. 5 additional subjects were removed from the modeling analyses because they were fit best by the bias variant of the model after PL or MA, which suggests that they were not using the reward probability or effort information in their decision-making for one or both conditions (computational modeling *N* = 91). Finally, 2–4 subjects were removed from the subjective ratings analyses due to missing data (subjective ratings ARCI Euphoria *N* = 94, DEQ Liking Drug *N* = 92).

## Procedure

### Orientation session

Participants first attended an orientation session to provide consent, complete questionnaires, and practice the study tasks. During orientation, participants were given instructions and completed practice trials of questionnaires and tasks. They also completed a full version of the EEfRT, and their performance on this administration was used as the baseline measurement of effort expenditure.

### Study sessions

Participants were instructed to refrain from using alcohol or pharmaceuticals except their usual caffeine and nicotine for 24 h before and 12 h after each study session. They were instructed to refrain from using recreational drugs (cocaine, amphetamine, cannabis etc.) for 48 h before the session, and 24 h after each session. They were told that they might receive alcohol, a sedative (e.g., Valium), stimulant (e.g., amphetamine or MDMA), or a placebo.

The two study sessions were each conducted from 9:00am to 1:00pm. Upon arrival for the sessions, participants provided breath (CAAlcosensor III, Intoximeters) and urine (CLIAwaived Inc, Carlsbad, CA) samples for alcohol and drugs, and women were tested for pregnancy (hCG assay; Abbott Laboratories, Abbott Park, IL). After these compliance tests, pre-drug physiological and subjective measures were obtained (see Measures). Then, participants ingested a capsule containing 20 mg MA or placebo under double-blind conditions. One hour later they completed behavioral tasks including the EEfRT. Physiological measures (heart rate and blood pressure) and self-report questionnaires were obtained at regular intervals (30, 90, 120, 150, 180, and 210 min) post-drug administration.

## Measures

### Subjective measures

Drug Effects Questionnaire - (DEQ; Fischman and Foltin 1991; Morean et al. 2013): The DEQ consists of questions on a visual analog slider scale (0–100) about the subjective effects of drugs. For this analysis we examined participants’ responses to the question “do you like the drug effect?”.

Addiction Research Center Inventory – (ARCI; Haertzen et al. 1963): The ARCI consists of 49 true/false questions measuring typical drug effects. For this analysis, we examined participants’ responses to the Morphine-Benzedrine group (ARCI-MBG) scale, which is thought to measure euphoric effects.

### Task

Effort Expenditure for Reward Task - (EEfRT; Treadway et al. 2009 ): EEfRT is a measure of effort-based decision-making. It consists of a series of trials on which participants select either a “hard” or “easy” option of a key-pressing task on a computer keyboard, for varying amounts of reward. The easy option requires pressing the “L” key 30 times with the right-hand pointer finger in 7 s and the reward is always $1. The hard option requires pressing the “R” key 100 times with the left-hand pinky finger in 21 s and the reward varies from $1.24-$4.21. Prior to task selection each trial, participants receive information about the reward value associated with the trial, as well as the probability (12%, 50%, or 88%) that they will win the reward if they complete the task.

### Physiological measures

Cardiovascular: Systolic pressure, diastolic pressure and heart rate were measured using a portable digital blood pressure monitor (LifeSource Model UA-787, A&D Medical, San Jose, CA).

## Data analysis

### EEfRT behavioral analysis

Based on previous research suggesting a relationship between baseline performance and drug effects on EEfRT (Soder et al. 2021 ), we used a median split to assign participants to high ( *N* = 48) and low ( *N* = 48) baseline effort subgroups derived from their orientation session EEfRT choices, calculated as a percentage of hard choices out of all trials. To assess drug-induced changes in effort expenditure in the full sample and between the subgroups, we modeled participant choices on EEfRT using the lme4 package in R with a generalized linear mixed-effects model (GLMM) with a logit link function for the binomial (hard/easy) outcome variable. We included fixed effects of drug (MA or PL), probability of reward (12%, 50%, or 88%), reward amount ($1.24-$4.21), baseline effort group (low or high), and sex (male or female). Post-hoc analyses were completed with IBM SPSS Statistics version 29.

### EEfRT computational modeling analysis

We fit a previously developed computational model of subjective value of reward (Cooper et al. 2019) to the EEfRT data, with subjective value (SV) referring to the perceived value of the hard and easy task choices on each trial. We fit the model to data for each participant for each session (MA and PL) using the following equation:

$${\mathit{SV=RP}}^ {\it{h}} - {\it{k}}{\mathrm{E}}$$

Where R is the objective reward presented on each trial, P is the probability of reward associated with that trial, and E is the effort required for each option (hard = 1, easy = 0.3). Free parameter *h* modifies subjective value based on participant choices and can be interpreted as probability sensitivity, while free parameter *k* captures individual differences in effort discounting (i.e., effort sensitivity). The subjective value for each trial choice is converted to a probability of selecting the hard task using a Softmax equation (Sutton and Barto 1998), with inverse temperature parameter *t* modulating sensitivity to value differences, thus influencing choice consistency:

$$\:\mathrm{p}\left(\mathrm{h}\mathrm{a}\mathrm{r}\mathrm{d}\right)\:=\:\frac{{\mathrm{e}}^{\mathrm{S}\mathrm{V}\mathrm{h}\mathrm{a}\mathrm{r}\mathrm{d}\bullet\:t}}{{\mathrm{e}}^{\mathrm{S}\mathrm{V}\mathrm{h}\mathrm{a}\mathrm{r}\mathrm{d}\bullet\:t}\:+\:{\mathrm{e}}^{\mathrm{S}\mathrm{V}\mathrm{e}\mathrm{a}\mathrm{s}\mathrm{y}\bullet\:t}}$$

We used the fmincon function in MATLAB 2024a to fit the subjective value model to data from each participant session (MA and PL) using maximum likelihood estimation. The parameters *k* and *h* were constrained to be between 0 and 10, while *t* was constrained between 0 and 100. All models were fit with 100 random parameter initializations to avoid local minima and were fit to the first 50 trials of each session. We also fit two other models to examine whether participants were incorporating all trial information (effort, probability, and reward amount) to make their decisions or using different strategies. See supplemental materials for details on these models. The best fitting model was determined by computing the Bayesian Information Criterion (BIC) for each model (Schwarz 1978).

We assessed drug effects on modeling results (*N* = 91) using mixed model ANOVAs for each mean free parameter estimate from the full model (*k*, *h*, and *t*) and change in model fit (∆BIC) with within-subjects factor of drug (MA or PL), and between-subjects factors of baseline effort group (low or high), and sex (male or female). Model parameter estimates were log-transformed prior to analysis due to non-normal distribution. For main effects and interactions where sphericity was violated, Greenhouse–Geisser corrected statistics are reported. Mixed model ANOVAs and post-hoc analyses were completed with IBM SPSS Statistics version 29.

### Subjective and cardiovascular measures

The effects of MA on subjective responses (ARCI-MBG/Euphoria; *N* = 94 and DEQ-Like Drug/Drug Liking; *N* = 92) and cardiovascular measures (heart rate and blood pressure; *N* = 96) were analyzed using the peak change in value from baseline at each session (MA and PL). We used mixed model ANOVAs for each peak change score with within-subjects factor drug (MA or PL) and between-subjects factors baseline effort group (low or high), and sex (male or female). Mixed model ANOVAs and post-hoc analyses were completed with IBM SPSS Statistics version 29.

### Drug-induced behavior and mood changes

To assess potential relationships between drug-induced changes in EEfRT performance and mood, we used drug change scores (MA-PL; ∆) on relevant variables. We used Pearson’s correlations (IBM SPSS Statistics 29) on drug change scores for subjective responses (∆ARCI-MBG/Euphoria and ∆DEQ-Like Drug/Drug Liking) and effort-based outcome variables (baseline effort and ∆hard task selection).

## Results

1. Overall and baseline effort subgroup EEfRT responses to probability and reward.

The main behavioral outcome variable from EEfRT was frequency of choosing the hard task. In the full sample, participants chose the hard option more often when the reward amount and/or probability was high. Main sex effects for the behavioral analysis are reported in the supplemental materials. The median frequency of hard task selection by the full sample of participants was 37.41%, which was used to generate the baseline effort subgroups. At the baseline session, the low baseline group chose the hard task on 34.58% (SEM 2.19) of trials and the high baseline group chose the task on 61.67% (SEM 2.43) of trials.

**Fig. 1:**
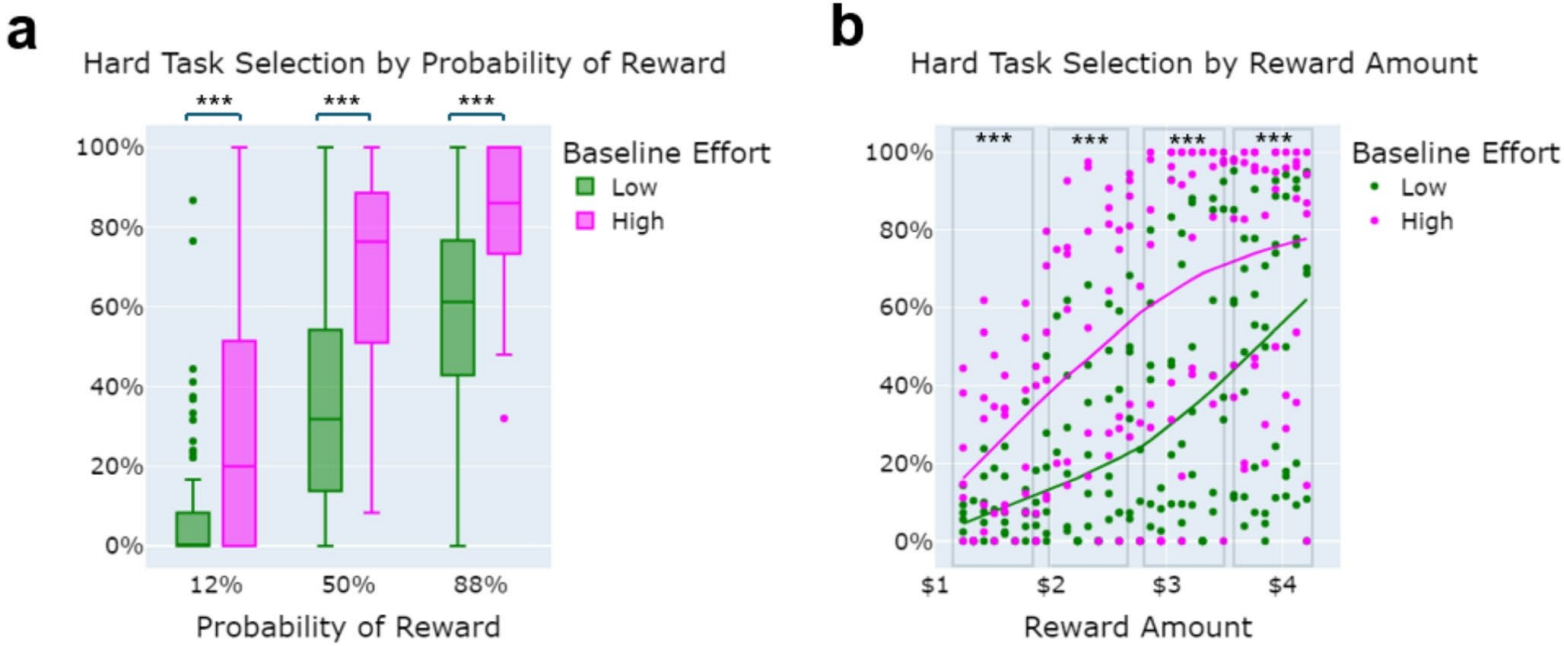
Performance on EEfRT during baseline session in participants divided into low and high effort selection (*N* = 48, 48). **a** Hard task selection by probability level separated by baseline effort group; **b **Hard task selection by reward amount separated by baseline effort subgroup (High (pink) vs. Low (green) baseline effort group *p* <.001***)

 Across all sessions, the low baseline effort group chose the hard task significantly less than the high baseline effort group overall, and at each probability level (Probability × Baseline Effort Group interaction, *B* = 0.325, SE = 0.066, *z* = 4.904, *p* <.001; post-hoc *t* -tests low vs. high baseline effort for *12* % - *t* (65.03) = −4.89, *p* <.001, *d* = −1.00; *50%* - *t* (94) = −7.80, *p* <.001, *d* = −1.59; *88%* - *t* (79.63) = −6.92, *p* <.001, *d* = −1.41; see Fig. 1 a). Additionally, across all three sessions, the baseline effort groups showed different patterns of responsiveness to reward. Reward magnitude was a continuous variable, but for post-hoc testing, we separated the variable into 4 bins based on quartiles of all available reward amounts (Bin1: $1.24–1.96; Bin2: $1.97–2.77; Bin3: $2.78–3.58; Bin4: $3.59–4.21). The low baseline effort group selected the hard task significantly less than the high baseline effort group across all bins (Reward Amount x Baseline Effort Group interaction, *B* = −0.273, SE = 0.051, *z* = −5.394, *p* <.001; post-hoc *t* -tests low vs. high baseline effort for Bin1: *t* (73.38) = −5.51, *p* <.001, *d* = −1.13; Bin2: *t* (94) = −7.76, *p* <.001, *d* = −1.58; Bin3: *t* (94) = −8.23, *p* <.001, *d* = −1.68; Bin4: *t* (94) = −7.41, *p* <.001, *d* = −1.51; see Fig. 1 b).

(2) Effects of MA on full sample and baseline effort subgroup EEfRT performance.

**Fig. 2:**
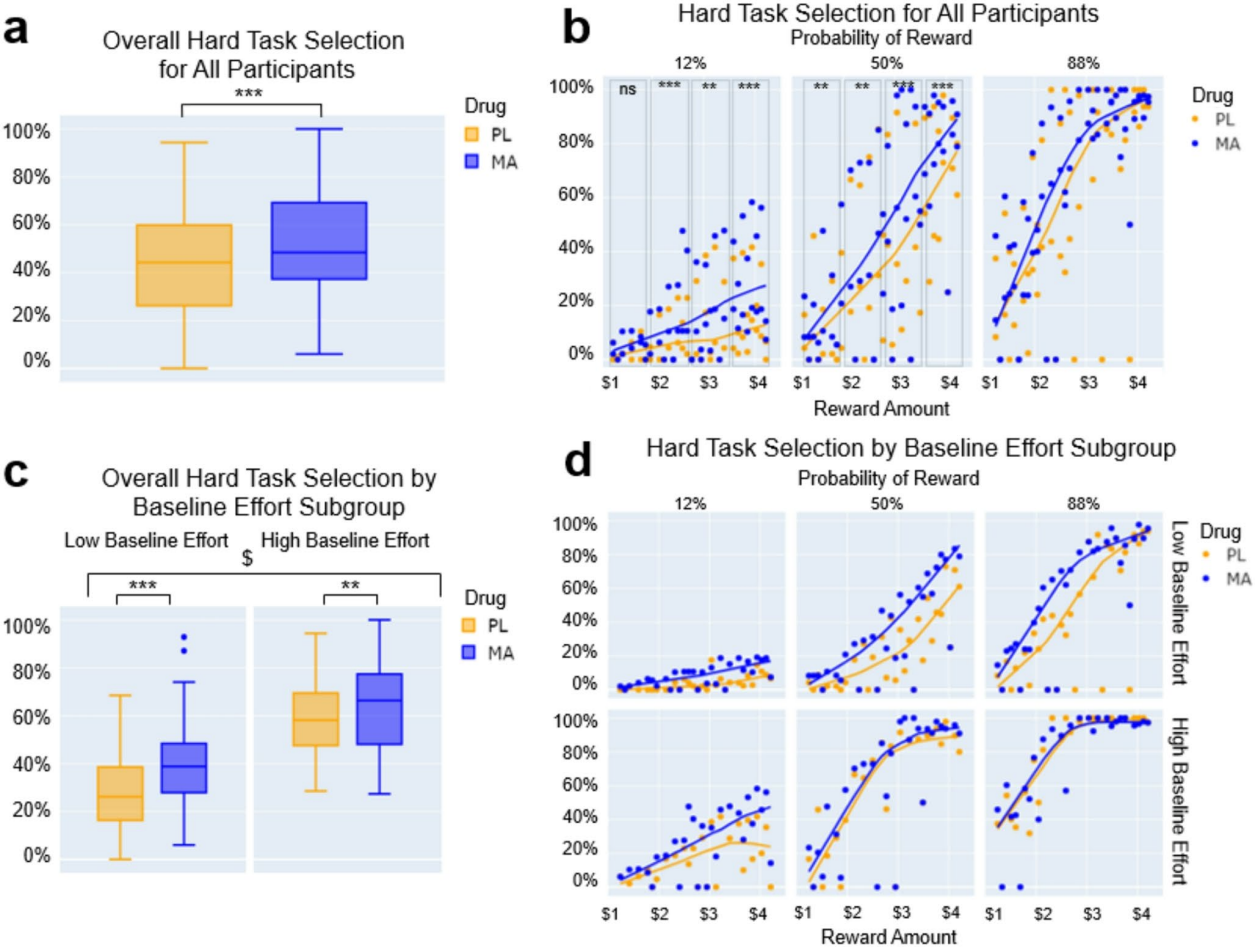
EEfRT performance after placebo (PL; yellow) and methamphetamine (20 mg; MA; blue) in all participants and participants divided by baseline performance. **a** Hard task selection for all participants by drug condition; **b** Hard task selection for all participants by drug condition split by reward bin and probability level; **c** Hard task selection for all trials by drug condition separated by baseline effort subgroup; **d** Hard task selection by drug condition split by effort subgroup, reward bin, and probability level (Drug x Baseline Effort Group interaction *p* <.001^$^, MA vs. PL *p* <.001***; MA vs. PL *p* <.005**)

Next, we looked at the effect of drug on task responses. In the full group, MA increased hard task selection overall (Drug main effect: *B* = −0.366, SE = 0.043, *z* = −8.43; *p* <.001; see Fig. 2a), particularly at low to medium probability levels. Post-hoc testing revealed that MA increased hard task selection at 12% probability in reward bins 2, 3, and 4, and all reward bins at 50% probability (Drug x Reward x Probability interaction: *B* = −0.159, SE = 0.058, *z* = −2.79, *p* =.012; post-hoc *t*-tests MA vs. PL for *12%* - Bin1: *t*(95) = 2.31, *p* =.012, *d* = 0.24; Bin2: *t*(95) = 3.60, *p* <.001, *d* = 0.37; Bin3: *t*(95) = 2.76, *p* =.004, *d* = 0.28; Bin4: *t*(95) = 4.18, *p* <.001, *d* = 0.43; and *50%* - Bin1: *t*(95) = 2.71, *p* =.004, *d* = 0.28; Bin2: *t*(95) = 2.90, *p* =.002, *d* = 0.30; Bin3: *t*(95) = 3.24, *p* <.001, *d* = 0.33; Bin4: *t*(95) = 4.31, *p* <.001, *d* = 0.44; see Fig. 2b). Statistical significance was assessed using Bonferroni correction for 8 comparisons (*p*_corrected_ = 0.006).

MA increased the overall frequency of hard task selection relative to placebo in both baseline effort subgroups when considering all trials per session. (Drug x Baseline Effort Group interaction: *B* = −0.178, SE = 0.043, *z* = −4.091, *p <*.001; post-hoc *t-*test MA vs. PL - low baseline effort group: *t*(47) = 5.228, *p* <.001, *d* = 0.76; high baseline effort group: *t*(47) = 2.898, *p* =.003, *d* = 0.42; see Fig. 2c). Additionally, the change in overall hard task selection (MA-PL) was significantly greater for the low baseline effort group compared to the high baseline effort group (post-hoc *t-*test high vs. low baseline effort group: *t*(94) = 2.538, *p* =.006, *d* = 0.52). While the Drug x Reward x Probability x Baseline Effort Group interaction did not reach significance, Fig. 2d displays the overall impact of low vs. high baseline effort on reward amount and probability responsiveness.

(3) Computational modeling of EEfRT.

**Fig. 3:**
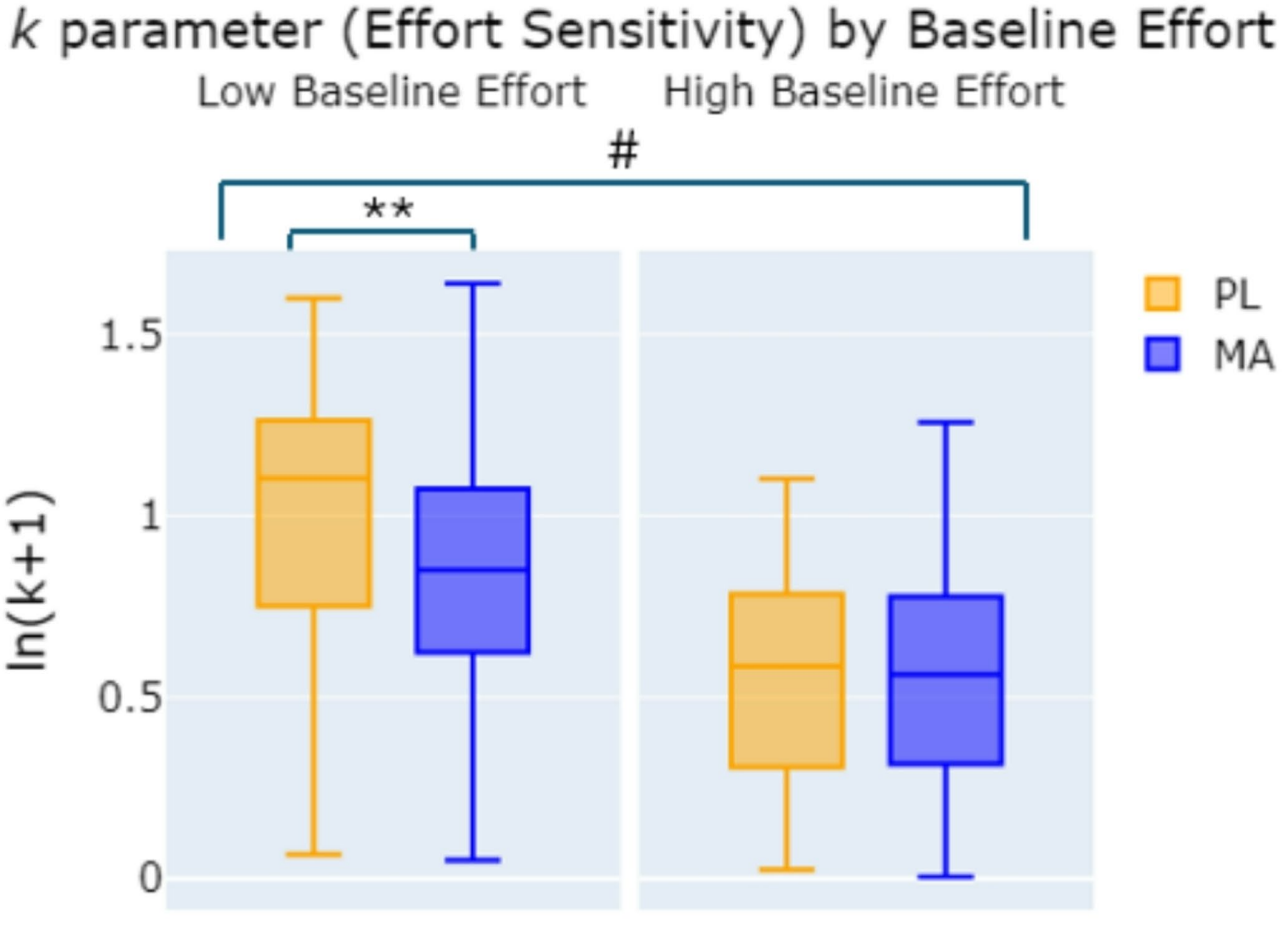
***k*** parameter after placebo (yellow) and methamphetamine (blue) in participants with low and high baseline EEfRT performance. Drug effect on *k* parameter in low and high baseline effort subgroups (Drug x Baseline Effort Group interaction *p* =.023^#^; MA vs. PL *p* =.002**)

We also examined the influence of both MA and baseline effort on each free parameter in the subjective value model and ∆BIC. MA (vs. PL) significantly reduced parameter *k* overall and in the low baseline effort group but not in the high baseline effort group. (Drug x Baseline Effort Group interaction: *F*(1,86) = 5.361, *p* =.023; post-hoc *t* test MA vs. PL for the low baseline effort group: *t*(44) = −3.114, *p* =.002, *d* = 0.32; and high baseline effort group: *t*(45) = −0.56, *p* =.478, *d* = 0.22; see Fig. 3). Sex effects for parameters *k* and *h* are described in the supplemental materials. There were no significant effects or interactions for parameter *t* or ∆BIC.

(4) Overall subjective and cardiovascular effects.

Across the whole sample, MA (vs. PL) increased ratings of euphoria and drug liking (Drug main effects: Euphoria - *F*(1,89) = 5.108, *p* =.026; Like Drug - *F*(1,87) = 7.011, *p* =.010). MA also increased systolic blood pressure (Drug main effect: *F*(1,91) = 96.36, *p* <.001), diastolic blood pressure (Drug main effect: *F*(1,91) = 52.41, *p* <.001) and heart rate (Drug main effect: *F*(1,91) = 86.42, *p* <.001). See Table 1 for a summary of subjective and cardiovascular effects.

(5) Subjective ratings in relation to EEfRT performance.

The effects of MA on euphoria and drug liking did not significantly differ between high and low baseline performers on EEfRT based on ANOVA results (See Table 1). Additionally, baseline effort as a continuous variable was not correlated with the drug change scores (MA-PL; ∆) for euphoria (baseline effort and ∆ARCI-MBG: *r =* -.40, *p* =.699) or drug liking (baseline effort and ∆DEQ-Like Drug: *r =* -.403 *p* =.682). Furthermore, in the whole sample, the drug change score for high effort selection during EEfRT was not correlated with drug change scores for euphoria (∆ARCI-MBG and ∆hard task selection: *r* =.007, *p* =.948) or drug liking (∆DEQ-Like Drug and ∆hard task selection: *r* = -.031, *p* =.768).

**Table 1:** Subjective and cardiovascular effects

	Subjective and Cardiovascular Effects						
	Total Sample		Low baseline effort		High baseline effort		Statistics (ANOVA Drug main effect)
	PL	MA	PL	MA	PL	MA	
Euphoria (ARCI MBG)	−0.50 (3.49)	6.57 (4.63)	−0.57 (3.88)	6.53 (4.63)	−0.43 (3.08)	6.62 (4.26)	*F*(1,89) = 5.11, *p* =.026
Like Drug	27.47 (27.75)	75.96 (26.02)	30.49 (27.68)	75.68 (29.68)	24.31 (27.79)	76.24 (21.90)	*F*(1,87) = 7.01, *p* =.010
Systolic Blood Pressure	−2.59 (13.58)	18.95 (14.57)	−3.46 (13.12)	20.83 (15.61)	−1.70 (14.12)	17.02 (13.31)	*F*(1,91) = 96.36, *p* <.001
Diastolic Blood Pressure	−4.16 (11.22)	8.99 (13.09)	−4.02 (11.59)	9.35 (14.86)	−4.30 (10.95)	8.62 (11.15)	*F*(1,91) = 52.41, *p* <.001
Heart Rate	0.51 (14.19)	20.91 (17.00)	−3.27 (12.36)	17.52 (16.56)	4.36 (15.01)	24.36 (16.91)	*F*(1,91) = 86.42, *p* <.001

Mean (and sd) change from baseline values after placebo and methamphetamine on subjective self-report measures and cardiovascular measures. Values are shown for the full sample and for low and high baseline effort groups. There were no significant interactions between baseline effort and outcome measures

## Discussion

This study examined the effects of MA on motivated, reward-related decision-making behavior and subjective reports of mood in 96 healthy adult volunteers. MA increased willingness to exert effort task in the full sample, and the increase in effort was more pronounced in participants with low effort at baseline. MA had its usual effects on euphoria and drug liking, but these mood effects were not related to task performance, either at baseline or after MA administration. Using a computational model to analyze the decision-making process, we found that MA decreased sensitivity to the perceived cost of effort in the low baseline performers but not in high baseline performers. This analysis extends our understanding of how baseline traits influence responses to stimulants and how behavioral effects of a stimulant drug are related to its subjective effects.

As reported previously in both humans and rodents (Cocker et al. 2012; Soder et al. 2021), we observed marked individual differences in baseline choice preference in options involving low effort and low reward versus high effort and higher reward. Some individuals showed hard task preference consistently across reward amount, probability of earning reward, and drug condition. It is possible that this trait of willingness to exert effort is related to variation in baseline DA levels, as has been reported with other behavioral traits (Cools and D’Esposito 2011). The finding that MA preferentially increased willingness to exert effort in those with low baseline effort is consistent with potential involvement of DA in EEfRT performance.

While MA particularly improved task performance for the low baseline effort group, we saw some increased selection of the hard option across the whole sample. This is also consistent with previous reports on amphetamine-type stimulants (Soder et al. 2021; Wardle et al. 2011). Closer examination of the data indicate that the increase in hard choice selection in the whole sample was seen primarily at low-medium rather than high reward probability levels. However, hard task selection increases with MA were unrelated to reward magnitude, in that drug-induced increases were similar across all reward amounts. For reasons that are not clear, Soder et al., found that amphetamine produced greater increases in hard task selection at low vs. high reward magnitudes. It is possible that the difference in DA-ergic drug selected (d-amphetamine vs. MA) or sample size between Soder et al. and this analysis influenced the reward magnitude responsiveness outcome.

We further examined *how* MA increased effort in the two baseline effort groups. We used a computational model of subjective value of reward to explore each participant’s sensitivity to perceived cost of effort and to probability of reward after drug and placebo. Evaluating these sensitivities allowed us to assess factors that affected each participant’s reward-related decision-making process and how those factors changed with MA administration. In the full sample, MA reduced the model parameter *k*, which represents sensitivity to effort cost, but did not affect parameter *h*, which represents reward probability sensitivity. This is consistent with previous work (Soder et al. 2021) and suggests that the drug’s effort-enhancing behavioral effects are primarily due to decreased effort discounting, rather than sensitivity to potential risk in receiving a reward. Furthermore, we found that this effect was driven by the low baseline effort group, and MA had no effect on effort discounting in the high baseline effort group. This finding is in line with the “inverted U-shape” DA hypothesis which proposes that there are optimal levels of DA for efficient cognitive functioning (Cools and D’Esposito 2011; Webber et al. 2021). It is possible that the low baseline effort reflects low DA function, and that MA remediates this deficit, contributing to a change in decision-making strategy.

We also examined the relationship between drug-induced changes in motivated behavior on the EEfRT and changes in mood and drug liking. Contrary to our expectations, greater preference for the hard task after MA was not correlated with MA-induced euphoria or drug liking. Additionally, the two baseline effort groups did not differ in subjective experiences after MA. This suggests that the typical positive subjective response to stimulants is not related to reward and effort valuation as measured by the EEfRT. This lack of correspondence raises important questions about the involvement of DA in the cognitive or mood effects of MA and suggests that there are notable dissociations between the two effects. The cognitive aspects and the mood aspects of reward processing (anticipation, receipt, learning, etc.) may involve varying levels of dopaminergic transmission or engage additional neurotransmitter systems, including noradrenaline and serotonin (Cruickshank and Dyer 2009). This is an important area of future research, to investigate the neurochemical mechanisms of the subjective experience, compared to the cognitive effects, of stimulant drugs.

### Limitations

There are several limitations to this study. First, the participants were healthy volunteers, who were relatively homogeneous regarding age and education, and, importantly, they were free of major psychiatric symptoms. It is not clear if the drug would have similar effects, either on the EEfRT task or in subjective experience in a more diverse sample, including those with symptoms of depression or ADHD. A second limitation is that we tested only a single dose of MA, making it difficult to separate sensitivity to the drug overall from true qualitative differences in the effects of MA. Third, we did not obtain any measure of DA function, so the involvement of neurotransmitter function is speculative. Future studies are needed using neuroimaging, such as PET to evaluate changes in DA levels, or fMRI to assess changes in affective and reward-based neural processing. Finally, some of the relationships we found were also affected by sex (see supplemental materials), but the limited sample size made this difficult to study.

## Conclusion

This analysis provides further insight into individual difference in baseline effort expenditure and its influence on the motivational and mood effects of stimulants. Specifically, in this healthy adult sample, a single, moderate dose of a stimulant drug increased willingness to expend effort and induced positive mood states in all participants. Notably, individuals with low baseline levels of effort experienced a larger increase in effort expenditure after MA, and only this group showed a drug-induced change in decision-making strategy. For these participants, the drug affected effort-based decision-making primarily by decreasing sensitivity to the perceived cost of effort (i.e. expending effort became less aversive), rather than altering sensitivity to reward probability. Importantly, neither baseline effort performance nor drug-induced increases in performance were related to the effects of the drug on positive mood. This suggests that different pharmacodynamic processes mediate reward valuation during EEfRT and mood alterations after MA. Our findings affirm the importance of considering baseline performance in assessing effects of drugs on motivational behavior and provide new information on the lack of relationship between drug-induced effects on cognition and mood.

## Electronic supplementary material

Below is the link to the electronic supplementary material.

Supplementary Material 1 (DOCX 94.7 kb)

## Data Availability

Data are available from the authors upon request.
