# Supplementary material for: Effects of methamphetamine on human effort task performance are unrelated to its subjective effects
Source: Psychopharmacology (Berl). 2025 Jul 8;243(1):199–209. doi: 10.1007/s00213-025-06853-4 (PMC12881056; doi:10.1007/s00213-025-06853-4)
Supplement: Supplementary file 1 — Supplementary Material 1 (DOCX 94.7 kb) [file 213_2025_6853_MOESM1_ESM.docx]

Effects of methamphetamine on human effort task performance are unrelated to its subjective effects

Evan C. Hahn, Hanna Molla, Jessica A. Cooper, Joseph DeBrosse, Harriet de Wit

**Supplemental Materials**

**Supplemental Table 1:** Demographics and drug-use history of study participants (*n* = 96), separated by participants who exhibited low or high effort at baseline.

| Demographic Categories |  | Low baseline Effort  n*(%) or mean (*SD*)* | High baseline Effort  n*(%) or mean (*SD*)* |
| --- | --- | --- | --- |
| Sex (M/F) |  | 21/27 (51/49%) | 27/21 (49/51%) |
| Age (years) |  | 24.2 (± 4.0) | 25.3 (± 4.0) |
| BMI |  | 23.7 (± 2.2) | 23.3 (± 2.8) |
| Education (years) |  | 15.5 (± 1.5) | 16.0 (± 1.4) |
| *Race/Ethnicity* |  |  |  |
| Asian |  | 9 (19%) | 8 (17%) |
| Black or African American |  | 4 (8%) | 1 (2%) |
| White |  | 28 (58%) | 33 (69%) |
| More than one race |  | 4 (8%) | 3 (6%) |
| Not reported |  | 3 (6%) | 3 (6%) |
| Hispanic |  | 9 (19%) | 9 (19%) |
| *Drug Use (mean uses past month)* |  |  |  |
| Caffeinated drinks/day |  | 1.0 (± 1.0) | 1.1 (± 1.0) |
| Alcoholic drinks/week |  | 3.1 (± 3.4) | 4.2 (± 4.4) |
| Cannabis uses/month |  | 3.4 (± 5.7) | 3.1 (± 5.7) |
| Daily nicotine users |  | 2 (4%) | 9 (19%) |
| *Lifetime stimulant users (ever used; N)* |  |  |  |
| Prescribed |  | 5 (10%) | 1 (2%) |
| Non-prescribed, medical |  | 9 (19%) | 8 (17%) |
| Recreational |  | 12 (25%) | 11 (23%) |
| *Other lifetime drug users (ever used; N)* |  |  |  |
| Cannabis |  | 45 (94%) | 44 (92%) |
| Opiates |  | 4 (8%) | 2 (4%) |
| Sedatives |  | 1 (2%) | 6 (13%) |
| Hallucinogens |  | 25 (52%) | 24 (50%) |
| MDMA |  | 9 (19%) | 13 (27%) |

**Subjective Value Model Selection**

We compared 3 models: a full subjective value model (see main text for details) which fits best for individuals who use effort, reward, and reward probability to guide their behavior, and two simpler model variants described in Cooper et al. 2019. The “reward SV” variant is a simplified version of the full subjective value model where parameter *h* is always 0, resulting in the equation:

$$SV = R-kE$$

A participant best fit by this model would only use reward and effort information to guide their behavior, neglecting probability. The “bias” model is the simplest model and includes a single free parameter that represents the probability of choosing the low effort option on each trial. Thus, the bias model is structured to best fit participants who make selections without incorporating reward and probability information. As most participants in this study were individually fit best by the full model under both drug conditions (as assessed by lowest Bayesian information criterion), we used the full model for analysis. We dropped participants if they were fit best by one of the other models in either condition. For the participants included in the analysis, we also calculated ∆BIC (BIC_bias_-BIC_SV_) for each participant for each condition, where a positive value indicates that including trial-wise information improved model fit.

**Sex Effects**

EEfRT Behavioral Analysis

Across the two study sessions, there was a main effect of sex, in which women selected the hard task less frequently overall than men did (Sex main effect, *B* = -0.462, SE = 0.166, *z* = -2.789,*p* = .006).

EEfRT Computational Modeling Analysis

There was an effect of sex on both primary subjective value model parameters. First, parameter *k* showed a significant change for the low baseline effort group but not the high baseline effort group, and that effect was influenced by sex (Drug x Baseline Effort Group x Sex interaction: *F*(1,86) = 6.006, *p* = .016, see Supplemental Fig. 1)*.* The low baseline effort group effect was driven by the female low baseline effort group, which had a significantly lower value for parameter *k* after MA administration (*t*(25) = -4.878, *p* < .001, *d* = 0.26). The female high baseline effort group (*t*(20) = 0.210, *p* = .418, *d* = 0.29), and low (*t*(18) = -0.087, *p* =.466, *d* = 0.34) and high (*t*(24) = -0.498, *p* =.311, *d* = 0.14) male baseline effort groups did not show significant drug effects for parameter *k*. Statistical significance was assessed using Bonferroni correction for 4 comparisons (*p*_corrected_=.0125).

There was also a sex effect for drug-induced change for parameter *h*, there was an effect of MA (Drug x Sex interaction: *F*(1,86) = 6.100, *p* = .015; see Supplemental Fig. 2). The women in this sample showed no difference between drug conditions (mean MA: 0.723 (0.494) vs. PL: 0.658 (0.371); *t*(46) = 1.039, *p* =.152, *d* = 0.43), while the men had a significantly lower parameter *h* value after MA (mean MA: 0.632 (0.306) vs. PL: 0.770 (0.418); *t*(43) = -3.017, *p* =.002, *d* = 0.

**Supplemental Fig. 1: k parameter after placebo or methamphetamine in females and males whose baseline performance was low or high.** Drug effect on *k* parameter separated by sex and baseline effort subgroup (Drug x Baseline Effort Group x Sex interaction *p*=.016^#^; MA vs. PL *p* = .001***)


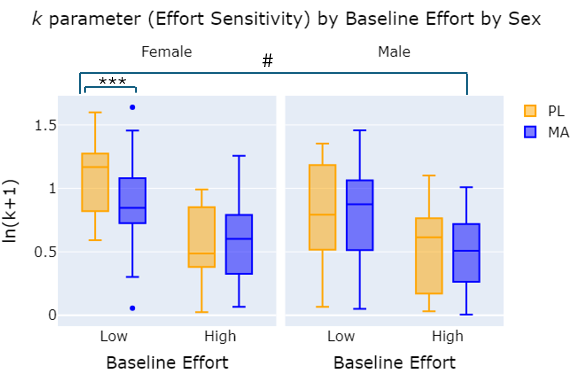


**Supplemental Fig. 2: Effects of methamphetamine vs placebo on h parameter separated by female and male participants.** Drug effect on *h* parameter separated by sex (Drug x Sex interaction *p*=.015^#^; MA vs. PL *p* = .002***)


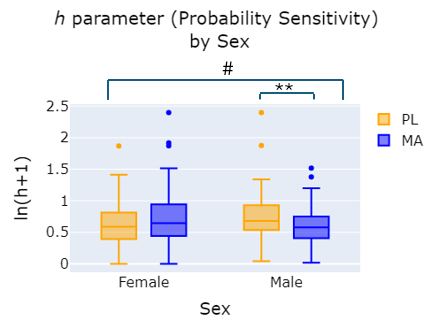


**References**

Cooper, J. A., Barch, D. M., Reddy, L. F., Horan, W. P., Green, M. F., & Treadway, M. T. (2019). Effortful goal-directed behavior in schizophrenia: Computational subtypes and associations with cognition. *Journal of Abnormal Psychology*, *128*(7), 710–722. https://doi.org/10.1037/abn0000443

Soder, H. E., Cooper, J. A., Lopez-Gamundi, P., Hoots, J. K., Nunez, C., Lawlor, V. M., Lane, S. D., Treadway, M. T., & Wardle, M. C. (2021). Dose-response effects of d-amphetamine on effort-based decision-making and reinforcement learning. *Neuropsychopharmacology: Official Publication of the American College of Neuropsychopharmacology*, *46*(6), 1078–1085. https://doi.org/10.1038/s41386-020-0779-8
